# Calcium Signaling Is a Universal Carbon Source Signal Transducer and Effects an Ionic Memory of Past Carbon Sources

**DOI:** 10.3390/ijms26052198

**Published:** 2025-02-28

**Authors:** Kobi Simpson-Lavy, Martin Kupiec

**Affiliations:** The Shmunis School of Biomedicine and Cancer Research, The George S. Wise Faculty of Life Sciences, Tel Aviv University, Tel Aviv 69978, Israel; kobisimpsonlavy@gmail.com

**Keywords:** calcium, yeast, saccharomyces cerevisiae, Snf1, carbon metabolism, maltose, sucrose, *ADH2*, *ZWF1*, AMPK

## Abstract

Glucose is the preferred carbon source for most cells. However, cells may encounter other carbon sources that can be utilized. How cells match their metabolic gene expression to their carbon source, beyond a general glucose repressive system (catabolite repression), remains little understood. By studying the effect of up to seven different carbon sources on Snf1 phosphorylation and on the expression of downstream regulated genes, we searched for the mechanism that identifies carbon sources. We found that the glycolysis metabolites glucose-6-phosphate (G6P) and glucose-1-phosphate (G1P) play a central role in the adaptation of gene expression to different carbon sources. The ratio of G1P and G6P activates analogue calcium signaling via the proton-exporter Pma1 to regulate downstream genes. The signaling pathway bifurcates with calcineurin-reducing *ADH2* (alcohol dehydrogenase) expression and with Cmk1-increasing *ZWF1* (glucose-6-phosphate dehydrogenase) expression. Furthermore, calcium signaling is not only regulated by the present carbon source; it is also regulated by past carbon sources. We were able to manipulate this **ionic memory** mechanism to obtain high expression of *ZWF1* in media containing galactose. Our findings provide a **universal** mechanism by which cells respond to all carbon sources.

## 1. Introduction

The availability of different carbon sources to an independent single-celled organism affects that cell’s metabolism, growth, and resistance to adversity. Although sensing and response to glucose or fructose (catabolite repression) have been extensively investigated (reviewed in [1]), how other carbon sources regulate cellular processes is poorly understood (except as a general no-glucose condition). Even within the “glucose response” body of research, a majority of research has focused on the activation of PKA by glucose to inhibit stress-responsive genes and the inhibition of Snf1 by glucose to explain glucose-repression of respiratory genes. Other elements of glucose signaling remain less characterized; such as the regulation by Snf1 of glucose-sensor activation of the Yck1/2 kinases [2,3] to regulate hexose-transporter expression and protein degradation [4,5,6]; the still-mysterious activation of the plasma membrane ATPase Pma1 pump, which consumes 50% of a glucose-grown cell’s ATP [7]; and calcium signaling (extensively reviewed in [8]). Furthermore, aside from the induction of hexose transporters by glucose via external sensing by Rgt2 and Snf3 [4,5,6], most known glucose-regulated signaling is repressive, and a mechanism of how glucose induces gene expression remains elusive.

The classical catabolite repression model of carbon-source gene regulation holds that glucose/fructose/mannose suppress metabolism and metabolism-related gene expression pertaining to other carbon sources, and this is regulated by PKA activation in response to glucose [9] and Snf1 (AMPK) activation in the absence of glucose [10]. Snf1 regulation of catabolite repression has been much studied, with a disproportionate focus on the Mig1 repressor, which is inhibited by Snf1 phosphorylation and disinhibited by Glc7-Reg1 dephosphorylation (e.g., [11,12]). However, much of the literature compares glucose to one other carbon source, usually galactose or glycerol, with little research comparing non-glucose carbon sources, which is somewhat surprising given that sucrose and maltose are the most important carbon sources commercially. Some carbon sources such as sucrose or glycerol have no known sensing mechanism, and although there are some transcription factors that respond to ethanol (e.g., Etp1 [13], Ert1 [14]), there is no known ethanol sensor. Only with respect to the relationship between acetate and ethanol do we understand the molecular mechanism: the acetate anion binds directly to the Haa1 transcription factor [15] and this activation of Haa1 represses *ADH2* expression to lower ethanol catabolism [16]. This also indicates the existence of more complex regulation within the set of non-glucose (fructose, mannose) carbon sources.

**Figure 1:**
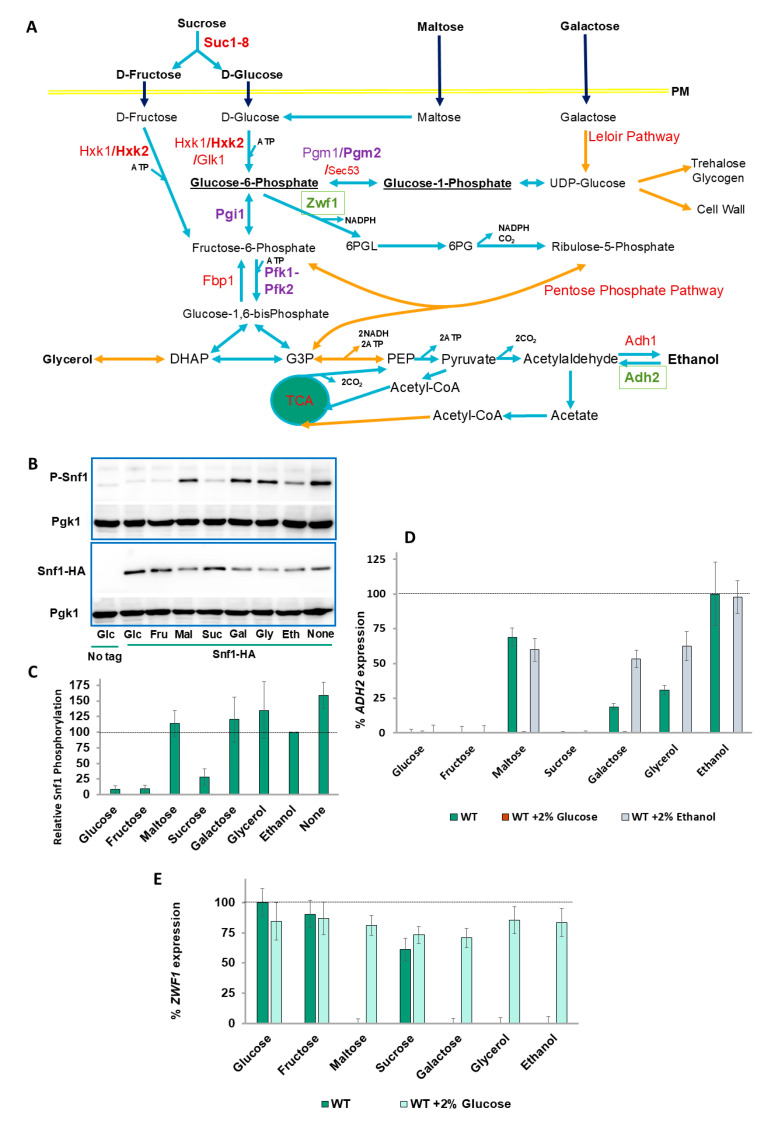
***ADH2* expression does not correlate with Snf1 activation.** (**A**) Schematic diagram illustrating carbon metabolic pathways of relevance to this article that are found in the V5 background. In **black bold** are carbon sources. **Black bold underlined** are glucose-1-phosphate (G1P) and glucose-6-phosphate (G6P). In **green** are the enzymes whose expression is investigated in the article. In **purple** are enzymes which are deleted in the strains used in the article. In **red** are other important enzymes and names of metabolic pathways. **Light-blue arrows** are single-step reactions, whereas **orange arrows** indicate multiple steps (omitted for clarity). (**B**) Glucose-grown cells were washed and then grown for 15 min in the indicated carbon source (**gl**u**c**ose (**Glc**), **fru**ctose, **mal**tose, **suc**rose, **gal**actose, **gly**cerol, **eth**anol or **none**), as described in Materials and Methods. Cells were processed and Western blots obtained, as described in Materials and Methods. The experiment was repeated **four (maltose) or five** times. Pgk1 is used as a loading control. P-Snf1 is Snf1 phosphorylated at T210. (**C**) Quantifications of Western blots of (**B**) were performed using ImageJ, version 2.0.0, as described in Materials and Methods. (**D**) *ADH2* expression in wild-type V5 cells was determined for the carbon sources indicated by b -galactosidase assay, as described in Materials and Methods. Expression rate is normalized to wild type in ethanol. N = 3. *T*-tests were performed, and all discussed differences are statistically significant (*p* < 0.05). (**E**) *ZWF1* expression in wild-type V5 cells was determined for the carbon sources indicated by β-galactosidase assay, as described in Materials and Methods. Expression rate is normalized to wild type in glucose. N = 3. *T*-tests were performed, and all discussed differences are statistically significant (*p* < 0.05).

Glucose is transported into a cell by seventeen hexose transporters (Hxt1-17), whereupon it is phosphorylated by Hxk2 (and also by Hxk1 and Glk1) [17]. There are three fates with respect to glucose-6-phophate (G6P) (Figure 1A): (a) isomerization by Pgi1 to fructose-6-phosphate followed by a second phosphorylation by phosphofructokinase (Pfk1-Pfk2) and glycolytic catabolism to pyruvate; (b) entry into the pentose–phosphate pathway by Zwf1 [18]; and (c) conversion to glucose-1-phosphate (G1P) by phosphoglucose mutases (PGMs: mainly Pgm1 and Pgm2) [19]. G1P is converted to UDP-glucose by Ugp1, which is the precursor of the storage carbohydrates glycogen and trehalose, and of the β-glycans and glucosamines that comprise the cell wall. Galactose is metabolized to G1P by the Leloir pathway and is converted to G6P by PGM to enter glycolysis (Figure S1A).

In *S. cerevisiae,* import of extracellular calcium is a response to depletion of internal calcium stores (primarily the vacuole) [20,21,22]. Calcium ions are imported into the cytoplasm from the external medium by the Mid1-Cch1 channel [23]. There are five known intracellular calcium pumps that sequester calcium into internal stores to maintain a cytoplasmic calcium concentration of 70–80 nM in glucose-grown cells [24] (with a total calcium concentration of 1.5–4 mM [24,25,26]). Calcium is released from these stores into the cytoplasm following exposure to glucose [27], cold stress [28], alkali stress [29], hypo-osmotic shock [22,30], toxic metals [28,31,32], and mating pheromones [33]. Exposure to glucose causes the release of calcium into the cytoplasm through the vacuolar Yvc1 channel [30] and other as yet unidentified channels [27]. This causes a rapid and transient elevation of cytosolic calcium levels (TECC) [34,35] that occurs 40–60 s after the addition of glucose [19]. The same pumps then re-sequester the calcium in the stores [35,36]. In contrast, a sustained calcium signal upon carbon source starvation is responsible for the initiation of autophagy [37].

The TECC response is dependent upon glucose import and phosphorylation by hexokinases [19], and a strain deficient for all hexose and maltose transporters fails to produce a TECC pulse upon exposure to glucose [19]. Moreover, an *hxk1Δ hxk2Δ glk1Δ* yeast strain, despite being capable of importing glucose, cannot phosphorylate it [17] or produce a TECC response upon exposure to glucose [38]. Following the addition of glucose, glucose-6-phosphate (G6P) and glucose-1-phosphate (G1P) levels increase after 30 s, preceding the TECC response (40–60 s after addition) [19]. The TECC response following glucose addition is delayed and diminished in *pgm2Δ* cells (which exhibit lower G1P levels) [19]. In summary, the balance between G1P and G6P regulates calcium accumulation: galactose-grown *pgm2Δ* cells (which exhibit high G1P) accumulate calcium; this can be suppressed by deletion of *PFK2* (thus increasing G6P) [39]. Inhibition of PGM activity by 10mM LiCl has the same effects on metabolite levels and calcium accumulation as *PGM2* deletion [40]. How the ratio between G6P and G1P is converted into a calcium signal remains to be determined. Once released into the cytoplasm, calcium acts as a second messenger to activate (via calmodulin) several kinases and the phosphatase calcineurin, whose activation of the Crz1 transcription factor has been extensively investigated [reviewed in [41]. A recent study has demonstrated calcium regulation of 30 proteins in *S. cerevisiae* [42].

In this work, we have examined up to seven different carbon sources for their effects on Snf1 phosphorylation and expression of downstream regulated genes. The rationale for choosing the carbon sources to be examined is as follows: glucose is the preferred carbon source of *S. cerevisiae*, which is fermented to ethanol, and whose presence represses utilization of other carbon sources [1]; fructose is metabolically and signaling-wise the same as glucose and phosphorylated by Hxk1 and Hxk2 to produce fructose-6-phosphate, but the *pgi1Δ* mutation prevents interconversion of glucose-6-phosphate and fructose-6-phosphate [43]; sucrose is a fermentable disaccharide of glucose and fructose that requires Snf1 for its metabolism and is hydrolyzed extracellularly by Suc2 [44]; maltose is a fermentable disaccharide of glucose that requires Snf1 for its metabolism [44]. Most laboratory strains such as those derived from S288C cannot utilize maltose [45]. However, maltose is commercially important; galactose is a fermentable monosaccharide requiring Snf1 for its metabolism [44]. It is converted to glucose-1-phosphate by the Leloir pathway [46]; glycerol is a non-fermentable carbon source requiring Snf1 for its metabolism [47] that enters glycolysis/gluconeogenesis as DHAP; and ethanol is a fermentation product that is respired via the glycoxlyate and TCA cycles and converted to glucose via gluconeogenesis. Ethanol requires Snf1 for its metabolism [48].

We find that calcium signaling provides a universal signaling mechanism in response to all carbon sources and regulates carbon source-dependent genes. In addition, calcium accumulation constitutes an ionic memory of prior carbon source exposure that modulates the response to fresh carbon source feeding.

## 2. Results

### 2.1. Snf1 Phosphorylation Levels Do Not Correspond with ADH2 Expression

Snf1 activity is required to switch from hexose fermentation to the respiration of poor carbon sources such as ethanol. By activating Adr1, the expression of *ADH2* [encoding alcohol dehydrogenase 2, which catalyzes the first step of ethanol catabolism (Figure 1A)], commonly used as a reporter for Snf1 activity, is induced. In the presence of glucose (or fructose), phosphorylation of Snf1 at T210 (which is usually taken as being indicative of Snf1 activation) is low (Figure 1B). After shifting the cells to other carbon sources for 15 min, there is a moderate amount of phosphorylation in the presence of sucrose (Figure 1B,C), despite Snf1 being required for growth with sucrose as the carbon source (Figure S1A). For other carbon sources (or in the absence of a carbon source), T210 phosphorylation is high, albeit less so in ethanol-containing media (Figure 1B,C). We also examined Snf1 phosphorylation after 3 h of growth in alternative carbon sources (in this experiment, native genomic Snf1 was detected in wild-type cells) (Figure S1B,C). After 3 h of growth in sucrose, Snf1 phosphorylation is similar to that of glucose-grown cells, probably due to invertase activity, and the Snf1 phosphorylation of maltose grown cells is lower in comparison to the 15-min time-point. Snf1 phosphorylation in galactose- and glycerol-grown cells is the same after three hours as ethanol- (or no carbon source-) grown cells (Figure S1B,C). In contrast, the expression of *ADH2* is markedly lower in galactose and glycerol compared with ethanol, and is absent entirely in sucrose-containing media (Figure 1D) [16].

### 2.2. ZWF1 Also Shows Differential Regulation by Carbon Source

We also investigated other carbon source-regulated genes. *ZWF1* encodes *S. cerevisiae*’s G6PDH, which catalyzes the first step of the oxidative pentose–phosphate pathway (Figure 1A), and its expression is dramatically upregulated following DNA damage by methylmethane sulfonate (MMS) and other oxidative stresses [49]. *ZWF1* expression in response to MMS requires glucose or fructose (with a lower expression of sucrose) and is markedly repressed by maltose, galactose, glycerol, and ethanol (Figure 1D). From a teleological perspective, this is logical since the substrate of Zwf1 is glucose-6-phosphate, so why produce this enzyme if the substrate is unavailable? However, this explanation does not provide a mechanism for how the different carbon sources are sensed. Adding 2% glucose to all poorer-carbon-source media restored *ZWF1* expression (Figure 1D), indicating that the inhibition of *ZWF1* expression is due to an absence of glucose (or fructose or sucrose) and not due to the presence of other carbon sources.

### 2.3. Snf1, PKA, and the SRR Pathways Do Not Regulate the Carbon Source Differential ADH2 and ZWF1 Expression Levels

Snf1 activity is required for expression of *ADH2* by causing the dephosphorylation of the Adr1 transcription factor (by an unidentified phosphatase). An Adr1^S230A^ mutant causes *ADH2* expression to occur in the absence of Snf1 [48]. However, expression of ADR1^S230A^ did not affect the expression of *ADH2* or *ZWF1* in the different carbon sources (Figure S1D,E). PKA is activated in *S. cerevisiae* by either Ras2 or Gpa2 sensing of glucose [50,51]. However, introduction of a constitutively active Ras2^G19V^ allele [50,51,52] does not affect *ADH2* or *ZWF1* expression in different carbon sources (Figure S1D,E). Another pathway that regulates genes that require glucose to be expressed is the SRR signaling pathway: glucose is sensed by Rgt2 and Snf3, relieving Rgt1 repression of (mainly hexose transporter) genes [4,5,6]. We examined the expression of *ADH2* (Figure S1F) and *ZWF1* (Figure S1G) in *rgt2Δ snf3Δ* cells. Inactivation of the SRR pathway does not affect the carbon source regulation of these genes.

Since there are no known signaling pathways that can discriminate between the different carbon sources, and indeed some carbon sources have no sensing mechanism (e.g., glycerol), we considered whether a metabolite could be responsible for these differences in gene expression, whereby the metabolite could potentially be a sensor for all carbon sources. Deletion of genes encoding specific glycolysis enzymes has previously been reported to cause accumulation of upstream metabolites when cells are grown on glucose (e.g., *TDH3* [53], *PFK2* [39]), or galactose (*PGM2* [39,54]).

### 2.4. A Balance of Hexose Metabolism Regulates Gene Expression

**Figure 2:**
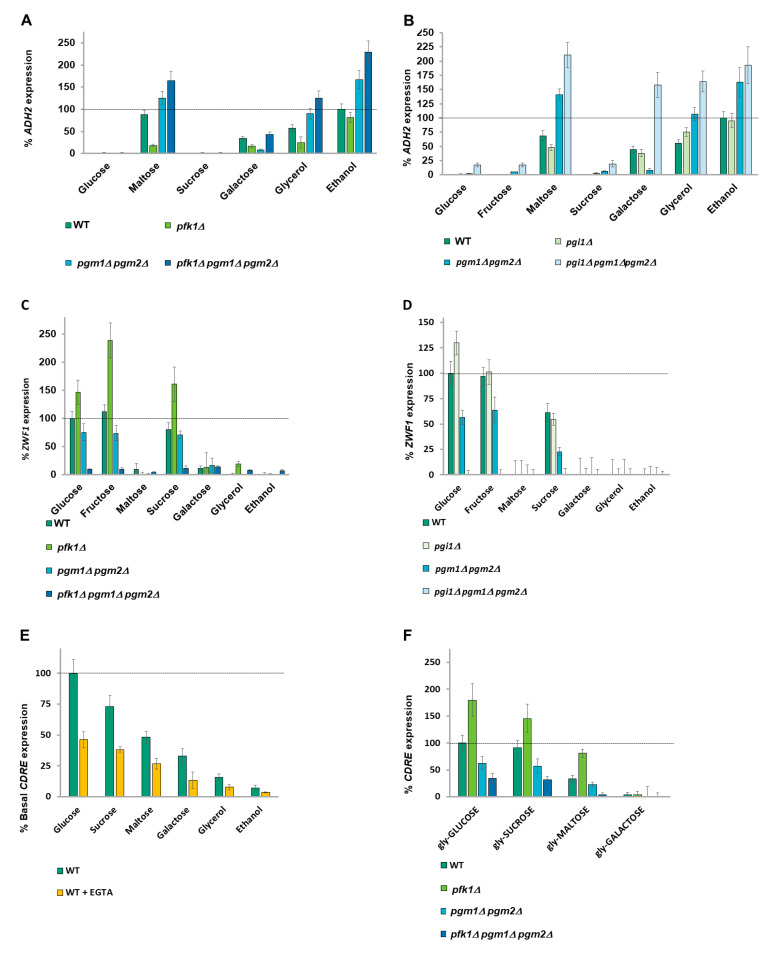
**Deletions of glycolysis genes affect *ADH2, ZWF1*, and *CDRE* expression.** (**A**,**B**) *ADH2* expression in V5 cells was determined for the carbon sources indicated by β−galactosidase assay. In (**A**), cells were grown overnight and diluted 10-fold in the morning with media containing 4% glucose; whereas in (**B**), cells were grown overnight and diluted 10-fold in the morning with media containing 2% fructose + 0.05% glucose due to the presence of a *PGI1* deletion [43] in some of the strains. Expression rate is normalized to wild type in ethanol. N = 3. *T*-tests were performed, and all discussed differences are statistically significant (*p* < 0.05). (**C**,**D**) *ZWF1* expression in V5 cells was determined for the carbon sources indicated by β-galactosidase assay. In (**C**), cells were grown overnight and diluted 10-fold in the morning with media containing 4% glucose; whereas in (**D**), cells were grown overnight and diluted 10-fold in the morning with media containing 2% fructose + 0.05% glucose due to the presence of a *PGI1* deletion [43] in some of the strains used. Expression rate is normalized to wild type in glucose. N = 3. *T*-tests were performed, and all discussed differences are statistically significant (*p* < 0.05). (**E**) The basal *CDRE* expression of wild-type V5 cells for the carbon sources indicated was determined by β-galactosidase assay. Expression rate is normalized to wild type in glucose. N = 3. *T*-tests were performed, and all discussed differences are statistically significant (*p* < 0.05). (**F**) *CDRE* expression upon addition of indicated carbon sources to glycerol grown V5 cells was determined by β-galactosidase assay. Expression rate is normalized to wild type in glucose. N = 3. *T*-tests were performed, and all discussed differences are statistically significant (*p* < 0.0.5).

An octameric complex of Pfk1 and Pfk2 [55] catalyzes the uni-directional glycolytic phosphorylation of fructose-6-phosphate by ATP to produce fructose-1-6-bisphosphate. The reverse reaction in gluconeogenesis is catalyzed by Fbp1 (Figure S1A). Single mutants of *PFK1 or PFK2* are viable in glucose (although with a lag phase during which they respire rather than ferment glucose [56] due to a throttling of glycolysis). However, both subunits are capable of catalyzing the phosphorylation of fructose-6-phosphate (F6P) [57]. We deleted *PFK1* since mutants of *PFK2* (but not of *PFK1*) also exhibit deficient Vma1 activity, leading to vacuolar alkalization and cytoplasmic acidification due to defective V-ATPase activity [58]. *pfk1Δ* cells exhibit a marked decrease in *ADH2* expression when maltose (a disaccharide made up of two glucose molecules), galactose, or glycerol are the carbon source compared to wild-type cells (Figure 2A), suggesting that the accumulation of F6P or an upstream metabolite is inhibitory to *ADH2* expression. To distinguish between F6P and G6P, we used a *pgi1Δ* strain [43] and observed that a blockage of glycolysis at the interconversion of G6P to F6P also inhibits *ADH2* expression (Figure 2B) when cells are grown with maltose as the carbon source (which enters glycolysis as intracellular glucose), indicating that the metabolite in question is either G6P or one of its precursors. We therefore decided to restrict another exit point of G6P by preventing its reversible conversion to G1P by phosphoglucomutases (PGM).

The production of G1P is essential for viability, with G1P being converted to UDP-glucose, which is, in turn, used for cell wall construction or production of storage carbohydrates. Pgm2 is the major phosphoglucomutase in *S. cerevisiae* [54]. Despite G1P being essential for viability, *pgm1Δ pgm2Δ* cells are viable in glucose media due to a low level of PGM activity provided by Sec53 [59]. The Leloir pathway converts galactose to G1P, which is converted to G6P by PGM to enter glycolysis (Figure S1A).

*pgm1Δ pgm2Δ* cells show an increase in *ADH2* expression in maltose, glycerol, and ethanol media, presumably due to reduced production of G1P (by Sec53) (Figure 2A,B). However, *ADH2* expression in *pgm1Δ pgm2Δ* cells is diminished in galactose (Figure 2A,B) since galactose metabolism produces G1P. Removal of both metabolic exits of G6P in a *pgm1Δ pgm2Δ pfk1Δ* or a *pgm1Δ pgm2Δ pgi1Δ* triple mutant results in a big increase in *ADH2* expression (Figure 2A,B), suggesting that it is the balance of G6P and G1P that is regulating *ADH2* expression, with higher G6P promoting expression.

### 2.5. ZWF1 Expression Is Also Regulated by Glucose-6-Phosphate and Glucose-1-Phosphate

*ZWF1* expression following 0.05% MMS treatment requires the presence of glucose, fructose, or sucrose as the carbon source, with low expression in maltose, galactose, and other poor carbon sources (Figure 2C,D). The expression profile of *ZWF1* is the inverse of *ADH2*, with a *pfk1Δ* (Figure 2C) or a *pgi1D* (Figure 2D) strain exhibiting higher *ZWF1* expression, suggesting that a metabolite above G6P promotes *ZWF1* expression. It is noted that whereas fructose causes very high *ZWF1* expression in a *pfk1Δ* strain (in which fructose-6-phosphate catabolism is restricted), fructose does not increase *ZWF1* expression in a *pgi1Δ* strain (in which fructose-6-phosphate catabolism is unimpeded) (compare Figure 2C,D). *pgm1Δ pgm2Δ* cells show lower *ZWF1* expression, suggesting that elevated G1P levels promote *ZWF1* expression, and blocking both phosphoglucomutases and either Pfk1 or Pgi1 activity reduces *ZWF1* expression to near zero (Figure 2C,D). This suggests that it is again the balance of G6P and G1P that is regulating *ZWF1* expression, with higher G1P promoting *ZWF1* expression and higher G6P repressing *ZWF1* expression. Thus, we conclude that the ratio of G6P to G1P is responsible for both the positive and negative regulation of carbon source-affected genes.

### 2.6. Calcium Signaling Is Regulated by All Carbon Sources

The pattern of regulation of *ADH2* and *ZWF1* expression by G1P and G6P levels bears a high resemblance to the regulation of calcium accumulation by these metabolites [39]. *FKS2* encodes a β-glucan synthase required for cell wall synthesis. Its promoter contains a 24bp calcium-dependent response element (CDRE) [60] which, when fused to a LacZ reporter, is used to determine Crz1 activity [27,60] and, by extension, nucleoplasmic calcium levels [27]. Basal *CDRE* expression was determined by growing cells in different carbon sources overnight and with dilution into fresh media containing the same carbon source for three hours. Basal *CDRE* expression is high in glucose and becomes progressively lower the further down the glycolysis of the carbon source is (Figure 2E). *CDRE* expression is sensitive to 1mM EGTA (which chelates external calcium [27,61], showing that this *CDRE* expression is due to calcium. **This suggests that calcium signaling generates a different analogue response for every carbon source**. We took glycerol-grown cells and added either 2% of different carbon sources (Glucose, Sucrose, Maltose, Galactose) or the same carbon sources together with 2% glucose. Similar to the basal expression experiment in Figure 2E, *CDRE* expression is dependent upon the carbon source added to glycerol-grown cells (Figure S2A). Further addition of 2% glucose restores expression of *CDRE* when co-present with all other carbon sources, with the *CDRE* response to glucose furthermore showing dose-dependency (as glucose was added either to 2% or to 4%) (Figure S2A).

### 2.7. Glucose Metabolites Signal to Calcium

Different carbon sources were added to glycerol-grown cells and the increase in *CDRE* expression was measured after 90 min. *pfk1Δ* cells exhibit a high increase in *CDRE* expression upon addition of either 2% glucose, 2% sucrose, or 2% maltose (but not 2% galactose), whereas *pgm1Δ pgm2Δ* cells exhibit about a one-third decrease in *CDRE* expression, with *pfk1Δ pgm1Δ pgm2Δ* cells showing an almost total abolishment of *CDRE* expression (Figure 2F). Treatment with the calcium chelator EGTA lowers *CDRE* expression by one-half (Figure S2B). Thus, the expression profile for *CDRE* resembles that of *ZWF1* and is opposite to that of *ADH2*, suggesting that the calcium release that is regulated by the G6P/G1P ratio is regulating expression (both positively and negatively) of *ZWF1* and *ADH2.*

### 2.8. Nucleo-Cytoplasmic Calcium Levels Both Positively and Negatively Regulate Carbon Source-Dependent Gene Expression

A number of calcium pumps regulate calcium availability in the cell. The Vcx1 and, to a lesser extent, Pmc1 calcium pumps remove calcium from the cytoplasm into the vacuole and are key for maintaining cytoplasmic calcium homeostasis [24]. Pmr1 pumps calcium into the Golgi [62] together with Gdt1 [63]. Cod1 pumps calcium into the endoplasmic reticulum [64].

**Figure 3:**
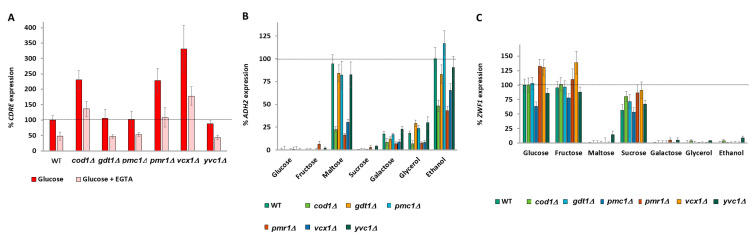
Deletions of calcium pumps regulate *CDRE, ADH2*, and *ZWF1* expression. *CDRE* (**A**), *ADH2* (**B**), and *ZWF1* (**C**) expression for V5 cells were determined as described in Materials and Methods. Expression rate is normalized to wild-type glucose (**A**,**C**) or to wild-type ethanol (**B**). N = 3. *T*-tests were performed, and all discussed differences are statistically significant (*p* < 0.05).

We screened the five calcium pumps for effects on carbon source-dependent gene expression. Knockout of either *COD1*, *PMR1*, or *VCX1* increases glucose-induced *CDRE* expression and this increase in expression is sensitive to the calcium chelator EGTA (1 mM). Knockout of *GDT1*, *PMC1*, or *YVC1* has no effect on glucose-induced calcium levels (Figure 3A). Knockout of *COD1*, *PMR1*, or *VCX1* lowers *ADH2* expression (Figure 3B), which is suppressed by prevention of calcium influx by the addition of 10 mM MgCl_2_ [27] (Figure S3A), showing that the phenotype is due to higher calcium levels in the nucleo-cytoplasm [27,65]. Knockout of *PMR1* or *VCX1* increases *ZWF1* (Figure 3C) expression, whereas knockout of *COD1* exhibits a milder increase in *ZWF1* with maltose as the carbon source (Figure 3C). These increases are also suppressed by 10 mM MgCl_2_ (Figure S3A,B). Furthermore, since deletion of any of these three calcium pumps affects gene expression, it is the nucleo-cytoplasmic calcium levels that are regulating gene expression rather than the calcium levels of a particular organelle. These three same calcium pumps have previously been shown to affect cytoplasmic calcium levels and the TECC response to glucose. Deletion of *VCX1* decreases vacuolar storage of calcium and raises cytoplasmic calcium levels, whereas deletion of *COD1* or *PMR1* increases the TECC release of calcium in response to glucose. Similar to our results, D’hooge et al. found that deletions of *GDT1* or *PMA1* have little effect on glucose-responsive calcium release [66]. Deletion of the vacuolar calcium-release channel Yvc1 did not affect *CDRE* expression, as expected [27], and did not affect *ADH2* or *ZWF1* expression (Figure 3A–C).

### 2.9. Raising Nucleo-Cytoplasmic Calcium Levels Suppresses Phenotypes Caused by Low G1P

We next decided to determine whether elevating nucleo-cytoplasmic calcium levels by knockout of *COD1* or *VCX1* would suppress the changes in gene expression observed in *pgm1Δ pgm2Δ* cells (*pgm1Δ pgm2Δ pmr1Δ* cells were not viable). Deletion of either *COD1* or *VCX1* restores expression of *CDRE* to at least WT levels (Figure S4A), suggesting that nucleo-cytoplasmic calcium levels are likewise restored in these cells. Deletion of either *COD1* or *VCX1* abolishes the increased *ADH2* expression of *pgm1Δ pgm2Δ* cells (Figure S4B) and suppresses the decreased *ZWF1* expression observed in *pgm1Δ pgm2Δ* cells (Figure S4C).

**Figure 4:**
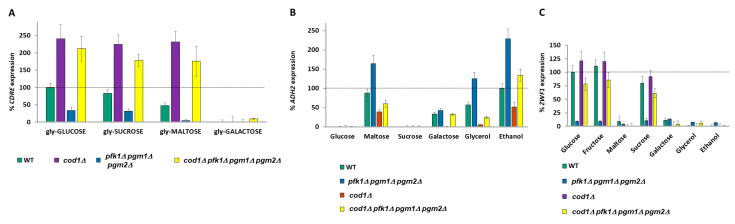
Deletion of *COD1* or *VCX1* suppresses phenotypes of *pfk1Δ pgm1Δ pgm2Δ* cells. *CDRE* (**A**), *ADH2* (**B**), and *ZWF1* (**C**) expression for V5 cells was determined as described in Materials and Methods. Expression rate is normalized to wild-type glucose (**A**,**C**) or to wild-type ethanol (**B**). N = 3. *T*-tests were performed, and all discussed differences are statistically significant (*p* < 0.05).

Since the phenotype of *pgm1Δ pgm2Δ* cells is sometimes rather mild (e.g., for *ZWF1* expression), we constructed *cod1Δ pfk1Δ pgm1Δ pgm2Δ* and *cod1Δ pgi1Δ pgm1Δ pgm2Δ* strains to determine whether raising nucleo-cytoplasmic calcium levels could suppress the severe phenotypes from blocking both metabolic exits of glucose-6-phosphate (Figure S1A). Further deletion of *COD1* restores nucleo-cytoplasmic levels of *CDRE* expression of *pfk1Δ pgm1Δ pgm2Δ* cells in glucose, sucrose, and maltose media to wild-type levels (Figure 4A). Further deletion of *COD1* likewise suppresses the increased *ADH2* (Figure 4B), and decreased *ZWF1* (Figure 4C) expression of *pfk1Δ pgm1Δ pgm2Δ* cells, with similar results for *ADH2* (Figure S4D) and *ZWF1* (Figure S4E) obtained when *PGI1* was deleted in lieu of *PFK1.* Although deletion of *COD1* alone does not drastically affect *ZWF1* expression (Figure 3C, Figure 4C and Figure S4C), deletion of *COD1* suppresses the low *ZWF1* expression phenotype of *pgm1Δ pgm2Δ* and of *pfk1Δ pgm1Δ pgm2Δ* cells. Jointly, our results demonstrate that increased nucleo-cytoplasmic calcium levels suppress the phenotypes of dysregulated G1P and G6P levels.

### 2.10. The G1P/G6P Ratio Signals to Pma1 Proton Pumping

Addition of glucose to media not only results in transient calcium influx into the nucleo-cytoplasm, but also activates the Pma1 proton pump [67], with Pma1 activity being required for calcium signaling but not vice-versa [67]. Surprisingly, the mechanisms by which both Pma1 and the TECC response are activated following glucose addition are unknown.

In a previous study, we genetically manipulated Pma1 activity using a non-inhibitable Pma1-*D*901 allele coupled with deletion of *HSP30* to elevate Pma1 activity [68] and an *EXP1* deletion to lower the amount of Pma1 at the plasma membrane. Here we continue to utilize *PMA1-Δ901 hsp30Δ* but have also introduced the *hrk1Δ ptk2Δ* double knockout, which cannot activate Pma1 in response to glucose [7]. We also utilize the *psg1Δ* knockout, which exhibits lower levels of plasma membrane-localized Pma1 [69].

**Figure 5:**
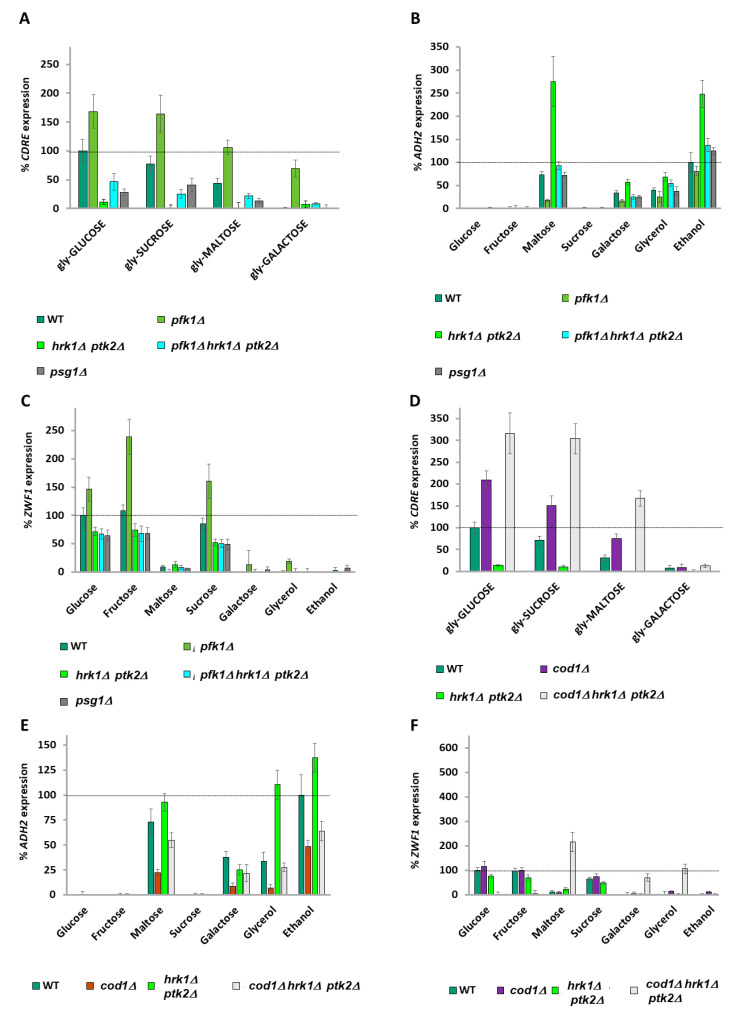
Manipulation of Pma1 activity suppresses *pfk1D* phenotypes. *CDRE* (**A**,**D**), *ADH2* (**B**,**E**), and *ZWF1* (**C**,**F**) expression for V5 cells was determined as described in Materials and Methods. Expression rate is normalized to wild-type glucose (**A**,**C**,**D**,**F**) or to wild-type ethanol (**B**,**E**). N = 3. *T*-tests were performed, and all discussed differences are statistically significant (*p* < 0.05).

Inhibition of Pma1 activity by deletion of *HRK1* and *PTK2* [7] decreases *CDRE* expression (calcium release) when glycerol-grown wild-type cells are fed glucose, sucrose, or maltose and lowers the high *CDRE* expression of *pfk1Δ* cells (Figure 5A). Lowering Pma1 levels at the plasma membrane by deletion of *PSG1* [69] likewise lowers *CDRE* expression. Inhibition of Pma1 by these methods increases *ADH2* expression (Figure 5B) and decreases *ZWF1* expression (Figure 5C). Moreover, it also suppresses the decreased *ADH2* (Figure 5B) and increased *ZWF1* expression (Figure 5C) of *pfk1Δ* cells. Hyper-activation of Pma1 (*PMA1-Δ901 hsp30Δ*) dramatically increases *CDRE* expression upon addition of glucose, sucrose, and even maltose (Figure S5A) and suppresses the low *CDRE* expression phenotype of *pgm1Δ pgm2Δ* cells (Figure S5A).

Pma1 hyper-activation (*PMA1-Δ901 hsp30Δ*) decreases *ADH2* expression and suppresses the high *ADH2* expression of *pgm1Δ pgm2Δ* cells (Figure S5B), indicating that G6P/G1P sensing precedes Pma1 for the regulation of *ADH2.* In contrast, reduction in Pma1 activity, whether by prevention of Pma1-tail phosphorylation (*hrk1Δ ptk2Δ*) or by decreasing its trafficking to the plasma membrane (*psg1Δ*), increases *ADH2* expression (Figure S5B), although *psg1Δ* has a more limited effect than prevention of Pma1-tail phosphorylation. Similarly to deletion of *COD1* (Figure 3C, Figure 4C and Figure S4C), hyper-activation of Pma1 (*PMA1-Δ901 hsp30Δ*) alone does not affect *ZWF1* expression but suppresses the low *ZWF1* expression of *pgm1Δ pgm2Δ* cells (Figure S5C). Together, these results suggest two possible models. The first is that the G6P/G1P ratio signaling is upstream of, and regulates, Pma1 activity. The second possibility is that the forced Pma1-induced changes to cytoplasmic pH overwhelm the metabolite-generated signal.

To differentiate between these possibilities, we determined whether upregulating calcium signaling could compensate for a lack of Pma1 activity. Additional deletion of the *COD1* calcium pump totally suppresses the *CDRE* expression defect of *hrk1Δ ptk2Δ* cells (Figure 5D). Deletion of *COD1* also lowers the *ADH2* hyper-expression phenotype of *hrk1Δ ptk2Δ* cells, though not to the same level as deletion of *COD1* in cells with functional Hrk1 and Ptk2 (Figure 5E). For *ZWF1* expression, a *hrk1Δ ptk2Δ cod1Δ* strain exhibits an inverted expression profile, with low expression in glucose and fructose media and yet very high expression in maltose, galactose, glycerol, and ethanol media (Figure 5F). Taken together, these results show that the phenotypes observed are due to calcium dysregulation in the absence of Pma1 activity.

### 2.11. Signaling Downstream of Calcium Bifurcates

**Figure 6:**
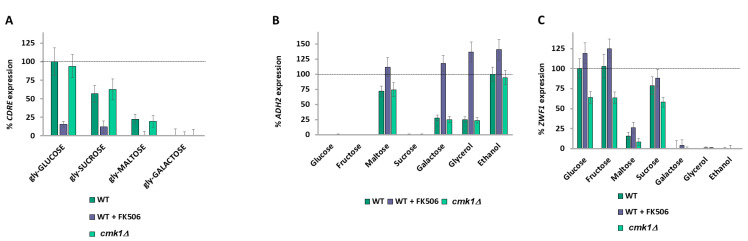
Signaling downstream of calcium is bifurcated. *CDRE* (**A**), *ADH2* (**B**), and *ZWF1* (**C**) expression for V5 cells were determined as described in Materials and Methods. Expression rate is normalized to wild-type glucose (**A**,**C**) or to wild-type ethanol (**B**). N = 3. *T*-tests were performed, and all discussed differences are statistically significant (*p* < 0.05).

Research into signaling downstream of calcium has been extremely biased towards the calcineurin phosphatase and its substrate Crz1, which it activates. Calcineurin inhibits Vcx1 pump activity [70] but Crz1 upregulates *PMC1* and *PMR1* transcription [60]. Other targets of calcium include the calmodulin-activated kinases (Cmk1, Cmk2, and Rck1). Treatment of glycerol-grown wild-type cells with 1 mM of the calcineurin inhibitor FK506 [60] just before addition of glucose, sucrose, or maltose drastically lowers *CDRE* expression, as expected (Figure 6A). Deletion of *CMK1* has no effect on *CDRE* expression, as expected (Figure 6A). Administration of 1mM FK506 immediately after washing cells and before resuspension in different carbon sources increases *ADH2* expression, with cells on media with maltose, galactose, and glycerol now having the same expression level as those in ethanol (Figure 6B). This suggests that in the absence of calcineurin signaling, the increased calcium levels (inferred by *CDRE* expression) of maltose-, galactose-, and glycerol-grown cells as compared to ethanol-grown cells (Figure 2E) no longer affect *ADH2* expression. Deletion of *CMK1* has no effect on *ADH2* expression (Figure 6B) but shows a marked decrease in *ZWF1* expression (Figure 6C). Thus, the downstream effector that regulates *ADH2* expression is calcineurin, whereas the downstream effector that regulates *ZWF1* is Cmk1.

### 2.12. Calcium Storage Provides a Memory Mechanism for Previous Meals

**Figure 7:**
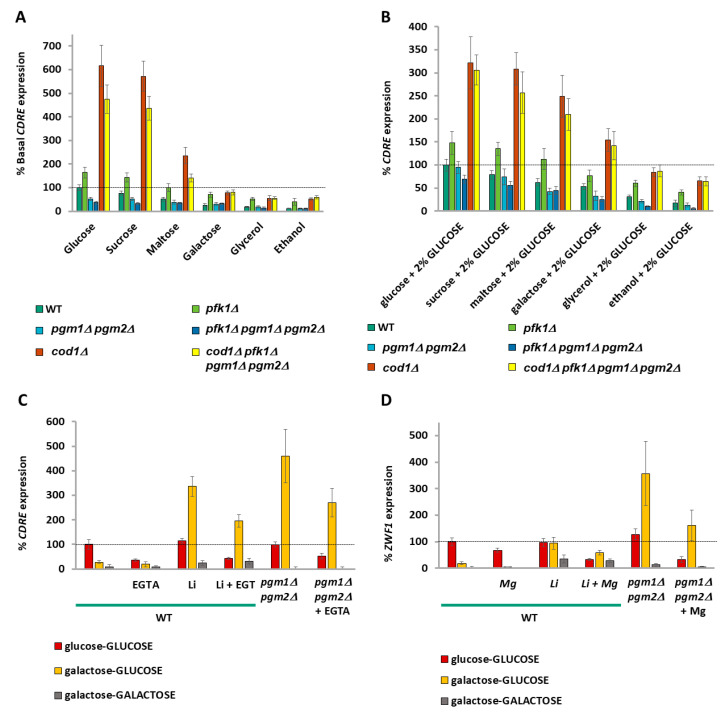
Calcium signaling serves as an ionic memory of prior meals. (**A**) The basal *CDRE* expression of V5 cells for the carbon sources indicated was determined by β-galactosidase assay. Expression rate is normalized to wild type in glucose. N = 3. *T*-tests were performed, and all discussed differences are statistically significant (*p* < 0.05). (**B**) Two percent glucose was added to the cells from (**A**) and *CDRE* expression was determined by β−galactosidase assay after an additional 90 min. Expression is normalized to wild type in glucose. Note that the *CDRE* expression of the glycerol-GLUCOSE condition is the normalizing condition for previous *CDRE* experiments. N = 3. *T*-tests were performed, and all discussed differences are statistically significant (*p* < 0.05). (**B**,**C**). *CDRE* (**C**) or *ZWF1* (**D**) expression in wild-type BY4741 cells following long-term treatment with 15mM LiCl or *pgm1Δ pgm2Δ* cells was determined by β-galactosidase assay, as described in Materials and Methods. Expression rates are normalized to wild-type glucose-GLUCOSE (**B**) or to WT glucose (**C**). N = 3. *T*-tests were performed, and all discussed differences are statistically significant (*p* < 0.05).

Growth in different carbon sources results in a differential level of basal cytoplasmic calcium (inferred by *CDRE* expression) in wild-type V5 cells (Figure 2E) as well as in wild-type BY4741 cells (Figure S6B). The basal *CDRE* expression is decreased in *pgm1Δ pgm2Δ* (and even more so in *pfk1Δ pgm1Δ pgm2Δ*) cells. Deletion of *COD1* dramatically elevates basal *CDRE* expression and suppresses the low basal *CDRE* expression of *pfk1Δ pgm1Δ pgm2Δ* cells (Figure 7A). As expected, the effects of *COD1* deletion are strongest when cells are grown with a carbon source that produces a lot of glucose phosphates (V5 cells have a growth defect on galactose, Figure S6A). We now wondered whether the calcium response to glucose would be the same irrespective of which carbon source the cells had been previously grown in. Upon addition of 2% glucose, cells previously grown in glucose or sucrose exhibit the highest increase in *CDRE* expression (calcium release), with cells pre-grown on galactose having 50% and other glycerol/ethanol pre-grown cells 20% of the *CDRE* expression of glucose pre-grown cells (Figure 7A). *pfk1Δ*, *pgm1Δ pgm2Δ*, and *pfk1Δ pgm1Δ pgm2Δ* cells all exhibit differential *CDRE* expression upon addition of 2% glucose depending upon the previous carbon source, although the amplitude of *CDRE* expression is greater than wild type for *pfk1Δ* cells across all prior carbon sources upon addition of 2% glucose and is lower than wild type for *pgm1Δ pgm2Δ* and *pfk1Δ pgm1Δ pgm2Δ* cells upon addition of 2% glucose (Figure 7B). Deletion of *COD1* strongly elevates *CDRE* expression upon addition of 2% glucose across all prior carbon sources and suppresses the low *CDRE* expression phenotype of *pfk1Δ pgm1Δ pgm2Δ* cells (Figure 7B). Remarkably, the degree of *CDRE* induction upon addition of 2% glucose correlates strongly with basal CDRE expression levels.

Since the V5 background has a growth defect on galactose and yet V5 *pgm1Δ pgm2Δ* cells are viable on galactose media (Figure S6A), we utilized the BY4741 background, which grows well on galactose media. We took the cells from Figure S6B, which had been grown overnight in different carbon sources, and determined the *CDRE* expression after addition of 2% glucose. Similar to V5 cells (Figure 7A), expression of *CDRE* in BY4741 cells is also dependent upon the prior carbon source, and the increases in *CDRE* expression are sensitive to EGTA (Figure S6C). Since the “basal” cells have had two exposures to their overnight carbon source (once upon seeding of cells and again when diluted in the morning and grown for 3 h), this suggests that exposure to carbon sources (especially glucose) has resulted in post-TECC pumping of calcium into the stores [66], and thus, prior exposure to glucose has led to an enhanced calcium release (as occurred in Figure 7B and Figure S6C), with calcium serving as an **ionic memory** of past carbon source exposures. This concept is now extended to all carbon sources, with the intensity of the response diminishing the further metabolically the carbon source is from glucose phosphates.

To show that increased calcium pumping due to a previous meal increases the intracellular levels of calcium and thus the TECC response, we decided to manipulate the cells to accumulate calcium by using the calcium hyperaccumulation phenotype of galactose-grown cells either with *PGM* deleted [39,54] or with PGM inhibited with 15 mM lithium chloride [40,71].

Since calcium accumulation in *pgm1Δ pgm2Δ* cells or wild-type cells treated with 15 mM LiCl is a very slow process [71], cells were grown in glycerol before being grown in either glucose or galactose media overnight in the presence of 15 mM lithium (and 1 mM EGTA), where indicated, before addition of an additional 2% glucose or galactose, as indicated, for determination of the increase in *CDRE* expression.

Lithium or knockout of *pgm1Δ pgm2Δ* did not affect *CDRE* expression upon addition of glucose to cells pre-grown in glucose, as would be expected since there is no increase in galactose-1-phosphate or calcium hyper-pumping. The addition of galactose-to-galactose pre-grown cells did not result in *CDRE* expression. However, lithium-treated wild-type cells or *pgm1Δ pgm2Δ* cells pre-grown in galactose have an approximately 10–15-fold increase in *CDRE* expression upon treatment with 2% glucose compared to cells not treated with lithium or possessing *PGM1 PGM2* (Figure 7C). Thus, we have divorced calcium accumulation from growth in glucose, using the calcium hyper-pump to create a false memory of prior food.

Does this apply to other genes? We adapted this protocol when examining *ZWF1* expression. Similar to *CDRE* expression, we found that overnight growth of lithium-treated or *pgm1Δ pgm2Δ* cells in galactose results in high *ZWF1* expression upon release of calcium by addition of 2% glucose to the media (Figure 7D). Thus, this memory mechanism also regulates other calcium-regulated genes.

## 3. Discussion

We have uncovered a molecular mechanism that allows cells to adapt their metabolic gene expression to match any carbon source. This system converts the presence of each carbon source into an analogue calcium output (best visualized in Figure 2E and Figure S6B). This mechanism does not override glucose-based catabolite repression, but within the set of permissive carbon sources, it regulates the degree of gene expression. For *ADH2* expression, this translates into Snf1 being active [48], and then calcineurin activity determines the precise level of expression, by analogy to a radio whereby, firstly, it must be switched on (by Snf1) and then the volume adjusted (by calcineurin). Hyper-activation of Adr1 by using an Adr1^S230A^ allele [48] does not affect the permissive carbon source regulation of *ADH2*. Inhibition of *ADH2* expression by increasing cytoplasmic concentrations of calcium, whether by metabolic (deletion of *PFK1* or *PGI1*) or calcium pump manipulation (deletion of *COD1*), indicates a role for calcium in the regulation of *ADH2* expression, with calcineurin activity being sufficient in galactose- and glycerol-grown cells to lower *ADH2* expression as compared with cells grown with ethanol as the carbon source. Although Crz1 is the best-characterized transcription factor working downstream from calcineurin, the *ADH2* promoter does not have a Crz1 binding site, suggesting that calcineurin communicates with other transcription factors. For *ZWF1* expression, Rpn4 is activated by MMS (or Yap1 by oxidative stress) [49] and then the extent of *ZWF1* expression is determined by Cmk1 activity.

We also found that this mechanism not only regulates glucose-repressed gene expression but also glucose-dependent gene expression, such as *ZWF1* induction following DNA damage. From a teleological perspective, it is logical for *ZWF1* to be expressed only when its substrate (glucose-6-phosphate) is present in sufficient quantity, and this mechanism of conversion of a G1P signal (which in wild-type cells *is* freely convertible with G6P) to a calcium level allows yeast to detect such carbon sources even in the absence of a specific sensor. Increasing cytoplasmic calcium levels, whether by metabolic or calcium pump manipulations, increases *ZWF1* expression. Calcium activates Cmk1, whose activity increases *ZWF1* expression above the basal sucrose level when cells are grown with glucose or fructose as the carbon source. In addition, when Pma1 phosphorylation (and activation) was prevented by deletion of *HRK1* and *PTK2* **and** cytoplasmic calcium levels increased by deletion of *COD1* (Figure 5F) or *VCX1*, an inverted phenotype occurred, with repression of *ZWF1* expression in normally permissive carbon sources (glucose, fructose, sucrose) and very high expression in normally non-permissive carbon sources (especially ethanol). Other genes (*CLN2*, *BCK2*, *CDC28*) have previously been found to be induced by glucose upon refeeding and are also regulated by accumulation of glucose phosphates, and, similarly to *ZWF1*, are not regulated by the SRR pathway [72]—perhaps these genes are also calcium regulated.

Most research into the calcium response to carbon sources has hitherto focused on glucose (e.g., [27]), although galactose has also been examined, usually in the context of Pgm2 inhibition [19]. In our hands, although glucose and sucrose addition to glycerol-grown cells resulted in high calcium signaling and permitted *ZWF1* expression while inhibiting *ADH2* expression, galactose resulted in a much-reduced calcium signal and did not permit *ZWF1* expression or totally repress *ADH2* expression. Furthermore, although the commercially important carbohydrates maltose and sucrose are both disaccharides of glucose and fructose, and both require Snf1 activity for their metabolism, although both are fermentable, their effect on expression of *CDRE, ADH2,* and *ZWF1* is dissimilar, with sucrose having the same profile as glucose and maltose having the same profile as galactose or glycerol.

An advantage of using a β-galactosidase reporter system is that the long half-life of the β-galactosidase protein results in a system that is sensitive to small changes. By growing cells overnight in different carbon sources, we were thus able to observe even small differences in the calcium response between galactose-, glycerol- and ethanol-grown cells (Figure 2E and Figure S6B) which are presumably unobservable by TECC measurements or GFP-based reporters. This demonstrates that **all** carbon sources have a demonstrable effect on basal levels of cytoplasmic calcium, thus broadening the scope of calcium signaling. The mechanism by which glycolysis metabolite levels transduce to a calcium signal is not known. Previous work revealed that it is the balance of G1P (pro-calcium) and G6P (anti-calcium) that regulates TECC [39], and presumably calcineurin activity [27] This work expands upon this model to include potentially all carbon sources, with those furthest from PGM activity exerting the least influence. This mechanism also provides a sensing mechanism for those carbon sources without specific sensors, such as sucrose, glycerol, or ethanol. The extent of gene regulation by a carbon source via calcium is also expanded beyond the traditionally investigated Crz1 targets to include both positive and negatively affected genes (*ZWF1* and *ADH2*). The full gamut of genes regulated by calcium in response to carbon source is likely to be much higher, since 32 carbohydrate metabolism genes are regulated by Cnb1 and Crz1 in response to media alkalization stress [73]. We also found comparable results to *ZWF1* expression for *HXT3* expression.

Furthermore, the response to 2% glucose is not uniform, but rather depends on the carbon source the yeast is growing in (Figure 7B and Figure S6B). This can arise since the pumping of calcium ions out of the cytoplasm is itself stimulated by glucose [66], and thus successive exposures to glucose would result in a feed-forward effect, with ever-increasing calcium pumping and release upon the next glucose exposure. This differential release of calcium upon exposure to glucose dependent upon the previous carbon source could be considered an **ionic memory** mechanism, whereby future responses to a carbon source are dependent upon past meals (Figure 7C,D). Other examples of protein-based memory systems for carbon sources include the sequestration of Std1 into puncta in glucose-grown cells, resulting in increased *ADH2* expression when cells are transferred to glycerol compared with galactose grown cells [74]; the Whi5 protein determining the time of G1 depending upon the environmental conditions of previous G1 stages [75]; or the accelerated expression of *GAL* genes upon transfer of cells from glucose to galactose if the cells had been previously exposed to galactose in the previous twelve hours [46].

Following the addition of glucose to cells, Pma1 is rapidly disinhibited by phosphorylation on the C-terminal inhibitory tail by the Ptk2 and Hrk1 kinases [7] (upon withdrawal of glucose, these sites are dephosphorylated by Glc7-Reg1 [76]). The mechanism by which glucose activates these kinases is not known. Pma1 activation is required for TECC response to glucose [67]. Therefore, we sought to integrate Pma1 activity into our schema of glucose-1-phosphate/glucose-6-phosphate somehow activating calcium signaling. We found that Pma1 hyper-activation (achieved by deleting *HSP30* and using a tail-less Pma1-*Δ*901 mutant [68]) suppresses the effects of altering glucose-6-phosphate/glucose-1-phosphate levels by the glycolysis mutants in *CDRE*, *ADH2*, and *ZWF1* expression (Figure 5A–C and Figure S5A–C), and that deletion of *COD1* in turn suppresses the effects of Pma1 inhibition (Figure 5D–F). This accords with previous work [67], but it is not clear from these data whether the G6P/G1P ratio is involved in Pma1 activation or whether Pma1 activity is simply a more significant regulator of calcium release. At each of these stages of metabolite balance, Pma1 activation, and calcium release, the molecular mechanism remains to be determined.

In mammalian cells, glucose stimulates calcium influx in brain tissue (glia, neurons, astrocytes) [77] and in the pancreas [78], whereas lactate (a poor carbon source that enters glycolysis as pyruvate) inhibits calcium entry into the cytoplasm in astrocytes and neurons and shifts metabolism towards respiration [79]. Lactate also inhibits calcium entry into pancreatic α-cells and glucagon secretion [80]. This is similar to the effect of carbon sources on calcium signaling in yeast that we have determined, and it is thus possible that a similar universal carbon source sensing role for calcium is preserved from yeast to man. In humans, calcium signaling is implicated in many disease states, such as diabetes [77,78,80], Alzheimer’s disease [81], and cancer [82].

To summarize, we have expanded the scope of calcium signaling to be a pan-carbon source sensor, with a different analogue response to each carbon source. We have also shown that a broader range of genes are regulated by calcium signaling than previously determined, both positively (*ZWF1*) and negatively (*ADH2*). The ratio of G6P/G1P is converted into a calcium signal, possibly via regulation of the cytoplasmic pH by Pma1. Furthermore, prior calcium signaling events serve as an **ionic memory** that impinges upon future carbon source events.

## 4. Materials and Methods

### 4.1. Contact for Reagent and Resource Sharing

Further information and requests for resources and reagents should be directed to and will be fulfilled by the Lead Contact, Martin Kupiec (martin@tauex.tau.ac.il). There are no restrictions on access to strains and plasmids.

### 4.2. Experimental Model and Subject Details

Experiments were conducted using *S. cerevisiae*. DH5a bacteria were used for plasmid propagation and standard procedures. Strains used are listed in Table 1; plasmids used are listed in Table 2. Additional information on materials appears as Table 3. All strains were related to the industrial champagne-derived yeast haploid V5 [83], except for strains used in Figure 7, which are in the BY4741a background [84]. Yeasts were transformed with DNA using the frozen lithium acetate method [84]. Where indicated, strains were transformed using plates containing 2% maltose instead of the usual glucose. Deletions were confirmed by PCR. Selective markers in plasmids were exchanged by gap repair [85]. Selective media was used throughout, comprising 6.7 g/L YNB, 1.5 g/liter amino acids and nucleotides, and a carbon source, as indicated. Drop-outs used were -Uracil and -Leucine -Uracil, where appropriate.

***ADH2* expression**: V5 yeast containing a plasmid expressing β-galactosidase from an *ADH2* promoter [48] were grown overnight at 30 °C in selective media containing 4% glucose, diluted ten-fold in the same selective media with 4% glucose, and then grown for another 3 h. When experiments involved cells deleted for *PGI1*, all strains were grown in media containing 2% fructose with 0.05% glucose [43]. Samples were taken for determining *ADH2* expression under repressive conditions (t = 0). Cells were washed three times with double-distilled H_2_O and then resuspended in selective media lacking any carbon source. 10 mM MgCl_2_ and 5 mg/mL FK506 (from a 20 mg/mL stock in 90% Ethanol/10% Tween-20) were added, where indicated. Cells were aliquoted into tubes, and the different carbon sources added to give a final concentration of 2% for glucose, fructose, sucrose, maltose, galactose, and ethanol and 3% for glycerol. Where indicated, an additional 2% glucose or 2% ethanol was also added (Figure 1D). Cells were grown for a further three hours and then *ADH2* expression determined by β-galactosidase assay (t = 3). Experiments were conducted in triplicate.

***ZWF* expression:** V5 Yeast containing a plasmid expressing β-galactosidase from a *ZWF1* promoter (358 bp upstream of the ATG) [49] were grown overnight at 30 °C in selective media containing 4% glucose, diluted ten-fold in the same selective media with 4% glucose, and then grown for another 3 h. When experiments involved cells deleted for *PGI1*, all strains were grown in media containing 2% fructose with 0.05% glucose [43]. Samples were taken for determining *ZWF1* expression under basal conditions (t = 0). Cells were washed three times with double-distilled H_2_O and then resuspended in selective media lacking any carbon source. MMS was added to give a final concentration of 0.05%. A total of 10 mM MgCl_2_ and 5 mg/mL FK506 (from a 20 mg/mL stock in 90% Ethanol/10% Tween-20) were added, where indicated. Cells were aliquoted into tubes, and the different carbon sources added to give a final concentration of 2% for glucose, fructose, sucrose, maltose, galactose, and ethanol and 3% for glycerol. Where indicated, an additional 2% glucose was also added (Figure 1E). Cells were grown for a further three hours and then *ZWF1* expression determined by β-galactosidase assay (t = 3). Experiments were conducted in triplicate. Note that for the experiment in Figure S1D, BY4741a yeast were used and cells grown overnight and the next morning in 2% ethanol, in order to prevent suppressor mutations occurring in the *rgt2Dsnf3D* cells.

***CDRE* expression:** For Figure 2E, Figure 7A and Figure S6B, wild-type V5 yeast containing a plasmid expressing β−galactosidase from 26 bp fragment of the *FKS2* promoter (CDRE motif) [60] were grown overnight at 30 °C in selective media containing 2% of the carbon source indicated (glycerol at 3%), with or without the calcium chelator EGTA (1 mM). Cells were diluted ten-fold in the morning into the same carbon sources (and more EGTA added where required) and grown for an additional 3 h before the basal *CDRE* expression was determined by β-galactosidase assay. Experiments were conducted in triplicate. Note that Figure 7A includes a repeat of Figure 2E (for wild-type cells). Figure S6B is the same experiment as Figure 2E but uses BY4741 cells instead of V5 cells. For Figure 2F, Figure S2A–C, Figure 4A, Figure S4A, Figure 5A,D, Figure S5A, Figure 6A, Figure 7B and Figure S6B, indicated V5 cells containing a plasmid expressing β-galactosidase from the 26bp fragment of the *FKS2* promoter (CDRE motif) [60] were grown overnight at 30 °C in selective media containing 3% glycerol + 0.1% glucose. A total of 1mM EGTA and 5 mg/mL FK506 (from a 20 mg/mL stock in 90% Ethanol/10% Tween-20) were present, where indicated. Cells were aliquoted into tubes and diluted in the morning into media containing 3% glycerol (no glucose) and grown for 3 h. Samples were taken for determining *CDRE* expression under basal conditions (t = 0). Indicated carbon sources (UPPERCASE) were added to 2% and the cells grown for 90 min before *CDRE* expression was determined by β−galactosidase assay (t = 1.5). Where indicated, an additional 2% glucose was also added (Figure S2A). For Figure 7B, cells from Figure 7A were treated with 2% glucose. For Figure S6C, cells from Figure S6B were treated with 2% glucose. Figure S6B,C use BY4741 cells instead of V5 cells. Experiments were conducted in triplicate. Note that this method cannot be used for *pgi1Δ* cells, so only experiments using *pfk1Δ* cells were performed to block glycolysis.

### 4.3. Figure 7C,D Experiments

Calcium accumulation in *pgm1Δ pgm2Δ* [39,54] or 15 mM LiCl-treated [40,71] cells is due to galactose-1-phosphate accumulation, which prevents growth in BY4741 cells [54] (Figure S6A). However, V5 cells are far less capable than BY4741 cells at utilizing galactose as a carbon source (Figure S6A), but unlike in the BY4741 background, V5 *pgm1Δ pgm2Δ* cells are viable in galactose. This deficiency in galactose metabolism was not rescued by deletion of *GAL80* (Figure S6A) encoding a repressor of GAL gene expression [59]. CouWe therefore conducted this set of experiments in the BY4741 background.

The aim of these experiments was to use either inhibition of deletion of phosphoglucomutase (PGM) activity to cause the cells to accumulate calcium when grown in galactose [40,71], which is then released by the addition of glucose.

For Figure 7C,D, BY4741 cells expressing β−galactosidase from either a *CDRE* (Figure 7C) or a *ZWF1* (Figure 7D) promoter were grown overnight at 30 °C in selective media containing 3% glycerol + 0.1% glucose. Cells were diluted 5-fold and grown for 6 h in media containing 3% glycerol (no glucose). Wild-type cells to be treated with 15 mM LiCl and to be grown in media containing 2% galactose or *pgm1Δ pgm2Δ* cells to be grown in media containing 2% galactose were diluted 4×. Other cells were diluted 50×. Cells were distributed into tubes and grown under indicated conditions (combinations of 2% glucose, 2% galactose, 15 mM LiCl, 1mM EGTA (Figure 7C)) for 16 h and the basal β−galactosidase activity measured (t = 0). For Figure 7D (*ZWF1* expression), 0.05% MMS was now added. The glucose-grown cells now had another 2% glucose added to them (glucose-GLUCOSE), and the galactose cells now had either 2% glucose (galactose-GLUCOSE) or 2% galactose (galactose-GALACTOSE) added. A total of 10 mM MgCl_2_ was added, as indicated (Figure 7D). After 90 min (Figure 7C) or 3 h (Figure 7D), the β-galactosidase activity was determined. Note that all the wild-type cells used in each experiment were from the same colony grown in 3% glycerol + 0.1% glucose. Experiments were performed in triplicate.

**β-galactosidase assay procedure:** Cell concentration was determined by reading 100 mL of cells at 595 nm. A total of 20 mL of cells was added to the β-galactosidase reaction mix for *ADH2* and *CDRE* and 50 mL for *ZWF1* expression determination (40 μL YPER (Pierce 78990), 80 mL Z-buffer (120 mM Na_2_HPO_4_, 80 mM NaH_2_PO_4_, 20 mM KCl, 2 mM MgSO_4_), 24 mL ONPG (4 mg/mL), 0.4 mL β-mercaptoethanol) and incubated at 30 °C for 15 min for *ADH2* and *CDRE* expression and for 30 min for *ZWF1* expression determination. Reactions were stopped by the addition of 50 mL 1.08 M Na_2_CO_3_. The Eppendorf tubes were centrifuged for 1 min at full speed to pellet the cell debris, and 200 mL of supernatant was removed and absorbance read at 415 nm using a microplate reader. Miller Units were calculated by the equation Miller Units = (1000 × A_415_)/(time × volume of cells × A_595_ − 0.055, where the A_415_ and A_595_ have been corrected for blanking and path length (final path length = 1cm). The Miller Units for t = 0 were subtracted from the Miller Units at t = 1.5 h or t = 3 h, and the difference was divided by the number of hours before being normalized against wild-type glucose for *CDRE* and for *ZWF1* expression and against wild-type ethanol for *ADH2* expression (defined as 100%). The t = 0 values were consistently very low for *ADH2* and for *ZWF1* expression, the latter requiring MMS treatment for expression to occur. Three biological replicates were measured. Error bars are +/− 1 standard deviation.

### 4.4. Western Blot Experimental Details

Indicated V5 *snf1Δ* cells with a plasmid expressing Snf1-2HA from a Snf1 promoter in pRS315 (or a wild-type strain with pRS315) (Figure 1B), or wild-type V5 cells (Figure S1B) were grown in selective media containing 4% glucose overnight. Cells were washed twice with 25 mL ddH_2_O and resuspended in selective media lacking any carbon source. Cells were aliquoted into glass tubes and the indicated carbon source was added to 2% (glycerol to 3%). Cells were grown at 30 °C for 15 min (Figure 1B) or for 3 h (Figure S1B) before processing.

Cells were harvested and protein solubilized in sample buffer by the method developed by the Kuchin group to prevent activation of Snf1 by centrifugation [87]. Cells were boiled for 5 min before treatment with 0.2 M NaOH. Sample buffer volume was adjusted to give equal OD for all samples. After running samples on 10% polyacrylamide gels, the proteins were wet transferred to nitrocellulose membranes. Antibodies used were mouse anti-Pgk1 (Abcam, Cambridge, UK) for a loading control, mouse anti-HA (Abcam, Cambridge, UK), mouse anti-polyhistidine (for detecting native Snf1 via the 13 polyhisitidine tract in the PKR of Snf1 [68]) (Abcam, Cambridge, UK), and rabbit anti-phospo-T172 (AMPK) (Cell Signaling Technology, Danbers, MA, USA), all at 1/1000 dilution. Since Snf1 runs at 72 kDa, the membrane was cut between 55 and 65 kDa, with the lower half used for Pgk1 and the upper for HA, polyHIStidine, or phospho-T172. Secondary antibodies were conjugated to HRP. Images were minimally processed using ImageJ, v. 2.0.0.

### 4.5. Serial Dilution Experiment Details

Freshly grown cells were taken from plates and resuspended in double distilled H_2_O. Following determination of OD_600_, the concentration of the yeast was normalized to OD4/mL. A total of 30 mL was taken and serially diluted ten-fold 5 times, and 5 mL final four dilutions spotted onto indicated plates. Photographs were taken after 2 days (Figures S1A and S6A), as indicated.

### 4.6. Quantification and Statistical Analysis

Statistical details are to be found in the figure legends. For β−galactosidase and Western blots, N represents the number of biological repeats. Error bars are one standard deviation. Western blots were quantified by finding the ratio of Snf1:Pgk1 (or phospho-Snf1:Pgk1) and then the ratio of [phospho-Snf1:Pgk1]:[Snf:Pgk1] for each experiment. Since the intensity of Pgk1 was not consistent over multiple experiments, these measurements were then normalized with wild-type ethanol, representing 100% phosphorylation. *T*-tests were performed on the data using Graphpad (https://www.graphpad.com/quickcalcs/ttest1/, accessed on 10 January 2025). All comparisons where a difference is reported are statistically significant (*p* < 0.05). All comparisons where a similarity is reported are statistically insignificant (*p* > 0.05) and have overlapping error bars.

## Figures and Tables

**Table 1:** Yeast strains.

Number	Name	Genotype	Source
V5 145	V5 WT	*ho ura3* *Δ*	Dequin lab [83]
V5 2	V5 leu2	*V5 leu2::hisG*	This project
V5 3	V5 snf1	V5 *leu2::hisG snf1::NATMX*	This project
V5 4	V5 pp	V5 *pgm1::NAT pgm2::HygBMX*	This project
V5 5	V5 pgi1	V5 *pgi1::KANMX*	Dequin lab [43]
V5 6	V5 pp pgi	V5 *pgi1::KANMX pgm1::NATMX pgm2::HygBMX*	This project
V5 7	V5 pfk1	V5 *pfk1::NATMX* (maltose)	This project
V5 8	V5 ppp	V5 *pfk1::NATMX pgm1::KANMX pgm2::HygBMX* (maltose)	This project
V5 9	V5 pp l2	V5 *leu2::hisG pgm1::NATMX pgm2::HygBMX*	This project
V5 10	V5 pp pgi1(hisG)	V5 *pgi1::hisG pgm1::NATMX pgm2::HygBMX*	This project
V5 11	V5 ppp (hisG)	V5 *pfk1::hisG pgm1::NATMXMX pgm2::HygB*	This project
V5 12	V5 cod1	V5 *cod1::KANMX*	This project
V5 13	V5 gdt2	V5 *gdt2::KANMX*	This project
V5 14	V5 pmc1	V5 *pmc1::KANMX*	This project
V5 15	V5 pmr1	V5 *pmr1::NATMX*	This project
V5 16	V5 vcx1	V5 *vcx1::KANMX*	This project
V5 17	V5 yvc1	V5 *ycv1::NATMX*	This project
V5 18	V5 pp cod1	V5 *pgm1::NATMX pgm2::HygBMX cod1::KANMX*	This project
V5 19	V5 pp vcx1	V5 *pgm1::NATMX pgm2::HygBMX vcx1::KANMX*	This project
V5 20	V5 pp pgi1 cod1	V5 *pgi1::hisG pgm1::NATMX pgm2::HygBMX cod1::KANMX*	This project
V5 21	V5 ppp cod1	V5 *pfk1::hisG pgm1::NATMX pgm2::HygBMX cod1::KANMX*	This project
V5 22	V5 ptk2 hrk1	*V5 hrk1::HygBMX ptk2::NATMX*	This project
V5 23	V5 psg1	V5 *psg1::KANMXMX*	This project
V5 24	V5 h30	V5 *leu2::hisG hsp30::KANMX*	This project
V5 25	V5 cod1 ph	*V5 hrk1::HygBMX ptk2::NATMX cod1::KANMX*	This project
V5 26	V5 vcx1 ph	*V5 hrk1::HygBMX ptk2::NATMX vcx1::KANMX*	This project
V5 27	V5 h30pp	V5 *leu2::hisG hsp30::KANMX pgm1::NATMX pgm2::HygBMX*	This project
V5 28	V5 cmk1	V5 *leu2::hisG cmk1:NATMX* (maltose)	This project
V5 29	V5 gal80	V5 *gal80::KANMX*	This project
V5 30	V5 pp gal80	V5 *pgm1::KANMX pgm2::HygBMX gal80::NATMX*	This project

	BY	*BY4741a his3* *D0 leu2D0 ura3D0 met15D0*	Lab stock
BY 1	BY snf1	*BY4741a snf1:HygBMX*	P. Cocetti [10]
BY2	BY rgt2snf3	BY4741a *rgt2::KANMX snf3::NATMX*	This project
BY 2	BY pp	*B*Y4741a *pgm1::KANMX pgm2::HygBMX*	This project
BY 3	BY gal80	BY4741a *gal80::KANMX*	This project
BY 4	BY pp gal80	*B*Y4741a *pgm1::KANMX pgm2::HygBMX gal80::NATMX*	This project

**Table 2:** Plasmids.

Number	Backbone	Contains	Original Backbone	Source
85	pRS 315	*prSNF1*::*SNF1-2HA*		Berger lab [86]
319	pRS 316	4x *prCDRE::*LacZ	pAMS366	Arino lab [60]
411	pRS 316	*prZWF1::*LacZ		Karpov lab [49]
467	pRS 315	*prRAS2::Ras2^G19V^*	pRS 316	Cannon lab [51]
488	pRS 316	*prADH1:ADR1^S230A^*	pRS 314	Young lab [48]
919	pRS 316	*prADH2*::LacZ		Young lab [48]
1367	pRS 315	*prPMA1::Pma1*D*901*		Kupiec lab [68]

β-galactosidase assay experimental details for Figure 1, Figure 2, Figure 3, Figure 4, Figure 5, Figure 6, Figure 7A,B and Figures S1–S6.

**Table 3:** Key resources.

Reagent or Resource	Source	Identifier
Antibodies		
mouse anti-HA	Santa Cruz	F7 Sc7392
rabbit anti-phospho T172 AMPK	Cell Signaling	40H9 2535
Mouse anti-Pgk1	Abcam	113687
Mouse anti-polyHIStidine	Abcam	18184
Goat anti-mouse HRP	Jackson	115035003
Goat anti-rabbit HRP	Jackson	111035144
Bacterial and Virus Strains		
DH5a	Lab stock	
Chemicals, Peptides, and Recombinant Proteins		
YPER	Pierce	78990
ONPG	Sigma	N1127
Na_2_CO_3_	Sigma	791768
Experimental Models: Organisms/Strains		
Please see Table 1		

## Data Availability

Data is contained within the article and Supplementary Materials.

## Supplementary Materials

The following supporting information can be downloaded at: https://www.mdpi.com/article/10.3390/ijms26052198/s1.
