# Supplementary figures and images for: Calcium Signaling Is a Universal Carbon Source Signal Transducer and Effects an Ionic Memory of Past Carbon Sources
Source: Int J Mol Sci. 2025 Feb 28;26(5):2198. doi: 10.3390/ijms26052198 (PMC11900981; doi:10.3390/ijms26052198)

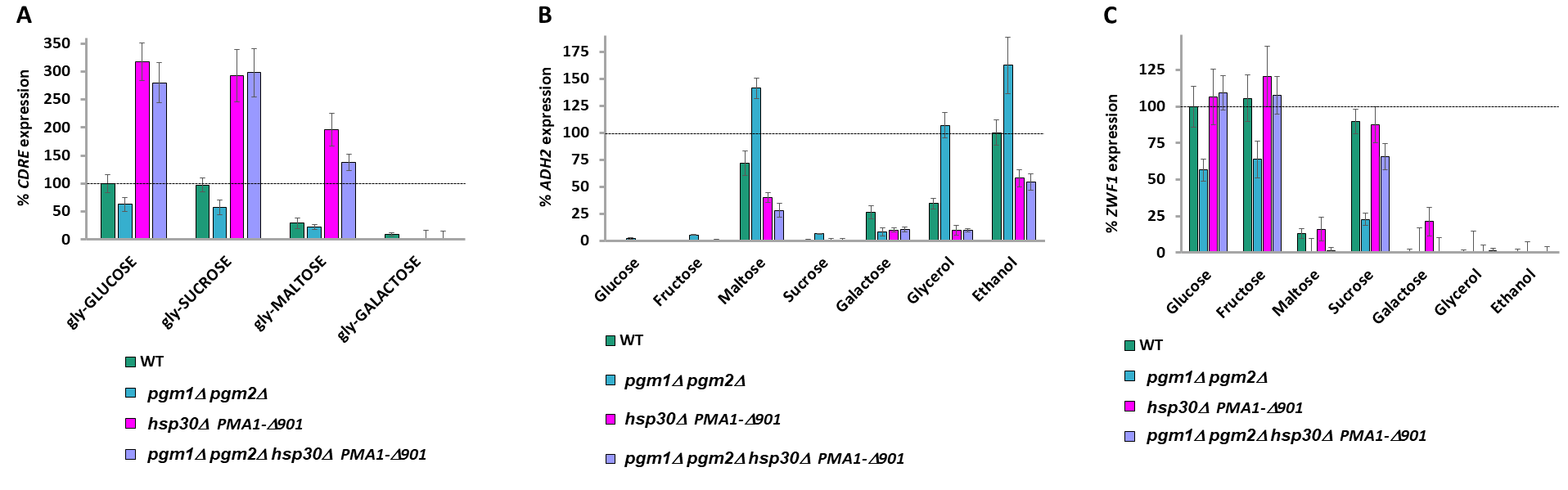

Supplement: Supplementary file 1 [file ijms-26-02198-s001.zip › Figure S5 (1).tif]

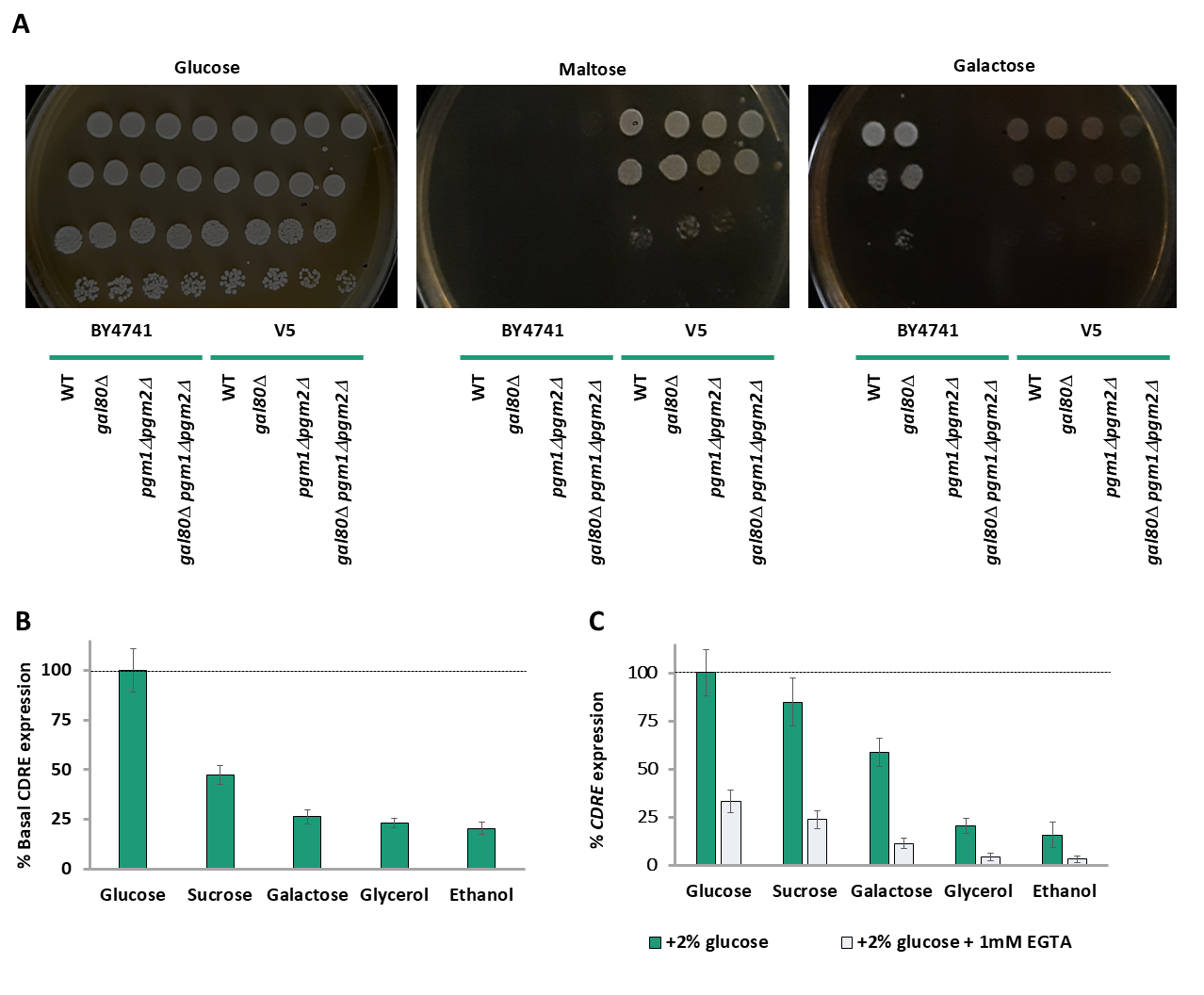

Supplement: Supplementary file 1 [file ijms-26-02198-s001.zip › Figure S6 (1).tif]

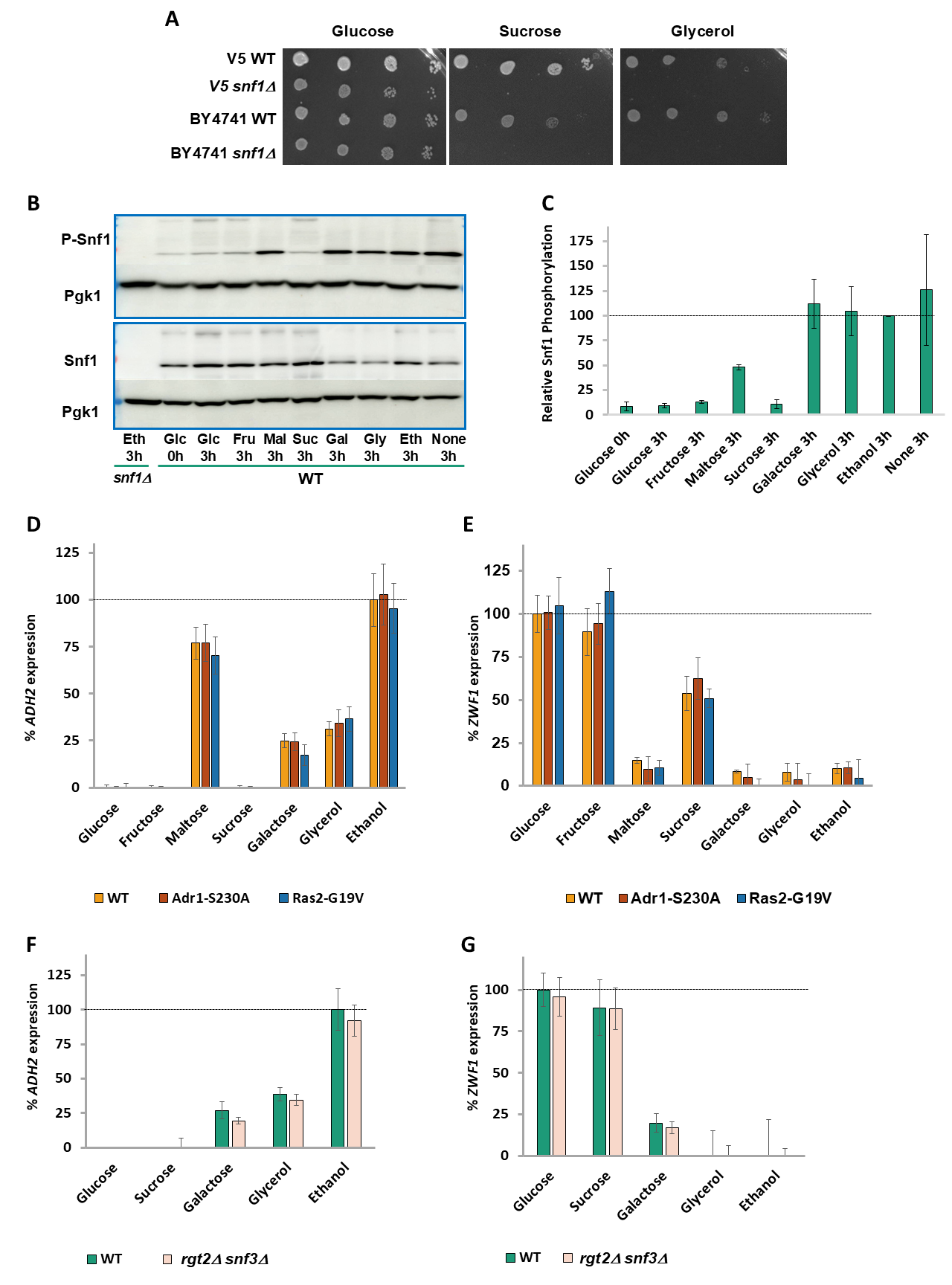

Supplement: Supplementary file 1 [file ijms-26-02198-s001.zip › Figure S1 (1).tif]

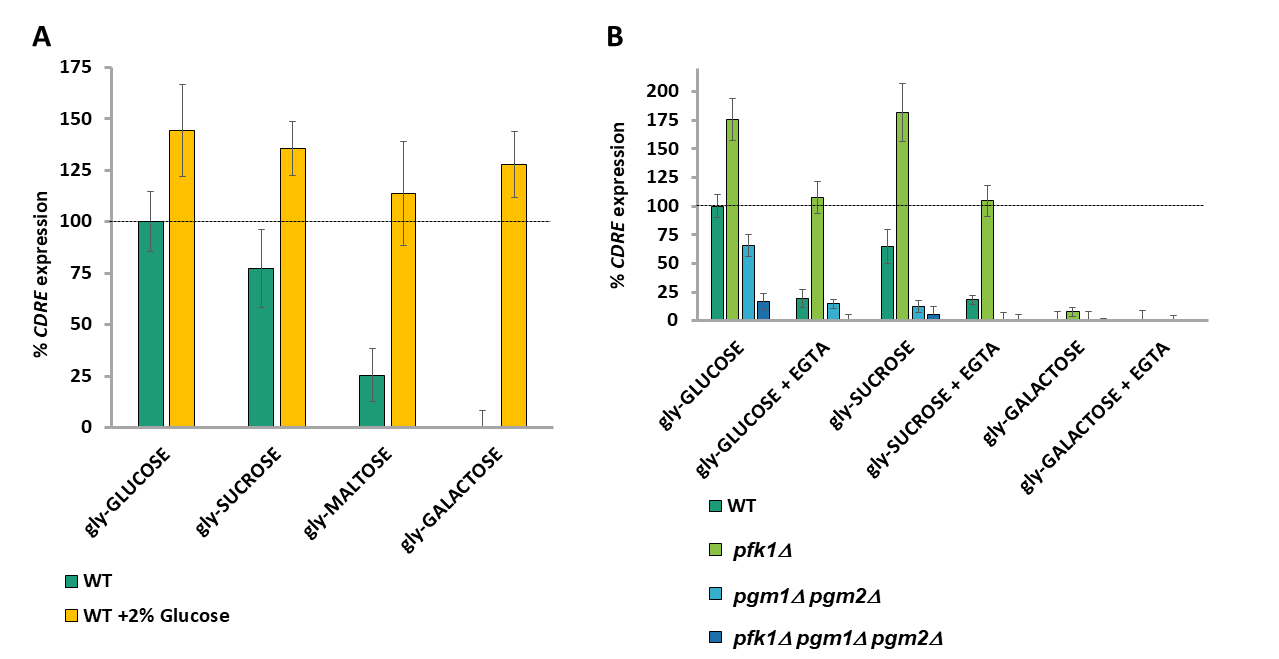

Supplement: Supplementary file 1 [file ijms-26-02198-s001.zip › Figure S2 (1).tif]

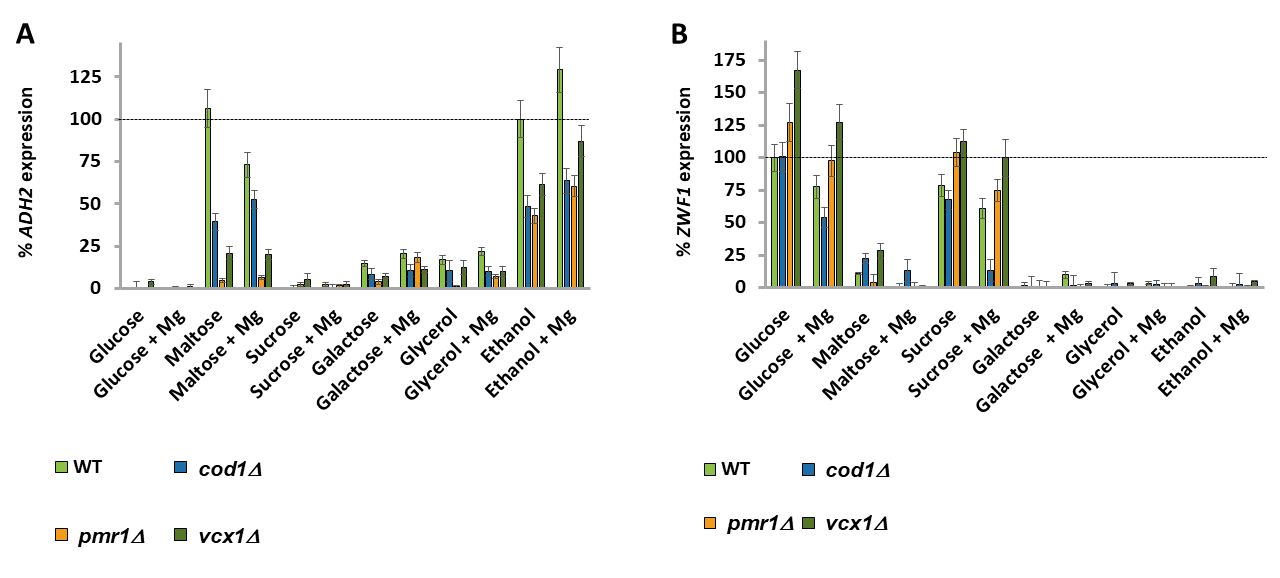

Supplement: Supplementary file 1 [file ijms-26-02198-s001.zip › Figure S3 (1).tif]

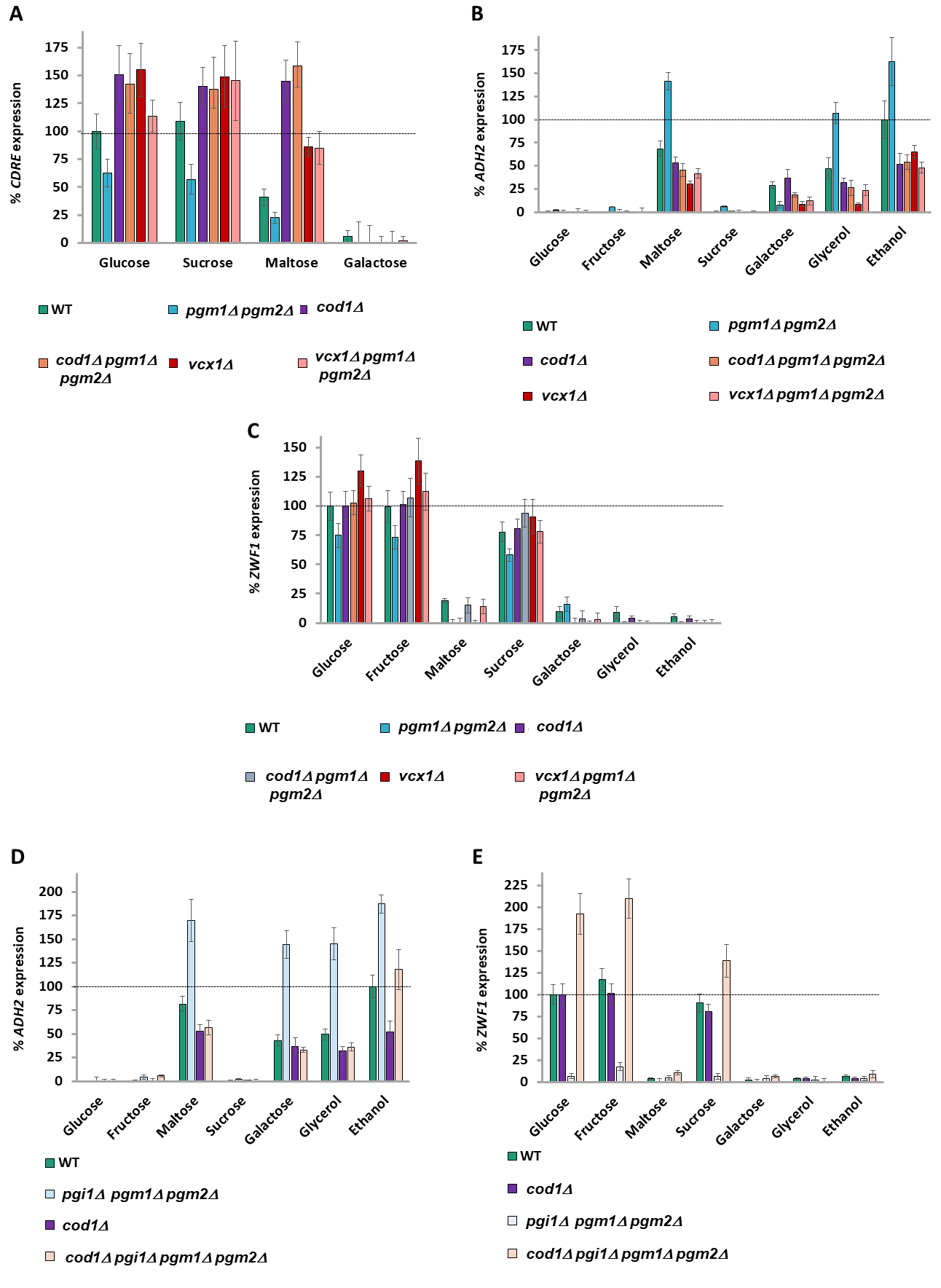

Supplement: Supplementary file 1 [file ijms-26-02198-s001.zip › Figure S4 (1).tif]
